# Regulation of anti-microbial autophagy by factors of the complement system

**DOI:** 10.15698/mic2020.04.712

**Published:** 2020-03-19

**Authors:** Christophe Viret, Aurore Rozières, Rémi Duclaux-Loras, Gilles Boschetti, Stéphane Nancey, Mathias Faure

**Affiliations:** 1 CIRI, Centre International de Recherche en Infectiologie, Team Autophagy Infection Immunity, Univ Lyon, Inserm U1111, Université Claude Bernard Lyon 1, CNRS, UMR5308, ENS de Lyon, F-69007, Lyon, France.; 2 Equipe Labellisée par la Fondation pour la Recherche Médicale, FRM.

**Keywords:** autophagy, complement, immunity, infection, bacteria, virus, homeostasis

## Abstract

The complement system is a major component of innate immunity that participates in the defense of the host against a myriad of pathogenic microorganisms. Activation of complement allows for both local inflammatory response and physical elimination of microbes through phagocytosis or lysis. The system is highly efficient and is therefore finely regulated. In addition to these well-established properties, recent works have revealed that components of the complement system can be involved in a variety of other functions including in autophagy, the conserved mechanism that allows for the targeting and degradation of cytosolic materials by the lysosomal pathway after confining them into specialized organelles called autophagosomes. Besides impacting cell death, development or metabolism, the complement factors-autophagy connection can greatly modulate the cell autonomous, anti-microbial activity of autophagy: xenophagy. Both surface receptor-ligand interactions and intracellular interactions are involved in the modulation of the autophagic response to intracellular microbes by complement factors. Here, we review works that relate to the recently discovered connections between factors of the complement system and the functioning of autophagy in the context of host-pathogen relationship.

## INTRODUCTION

Macro-autophagy, or autophagy, is a highly conserved cellular process that directs cytosolic components to endo-lysosomal compartments for degradation and recycling. Autophagy is required for the proper adaptation of cells to changes in their microenvironment and therefore, for cell homeostasis [1, 2]. Autophagy is constantly at work under physiological conditions, it ensures the disposal of aggregated/malformed macromolecules and organelles with signs of senescence or dysfunction. The actual removal involves the encapsulation of the targeted cargo into double-membrane vesicles named autophagosomes that undergo fusion with lysosomal vesicles to form autolysosomes where the effective degradation occurs. Because autophagy greatly contributes to the maintenance of cell homeostasis, its proper functioning is crucial for all biological functions and defective autophagy necessarily translates into various pathological manifestations [3]. Autophagy was also found to be instrumental to resist intracellular invasion by many different microorganisms thus representing an important component of cell autonomous defense mechanisms. The elimination of cytosolic microbes through the autophagic pathway is referred to as xenophagy [4]. During this process, the autophagy machinery can target components of replicating microbes for degradation but also entire microorganisms such as *Group A Streptococcus* (GAS) or *Salmonella enterica Serovar Typhimurium* (*S. Typhimurium*) [5, 6, 7]. As viruses are, by definition, intracellular parasites, autophagy is often mobilized to oppose viral invasion/multiplication both *in vitro* [8, 9, 10] and *in vivo* [11, 12, 13], including during the earliest steps of virus-host cell interactions [14]. Perhaps, the strongest indication that autophagy represents a highly efficient host defense mechanism to oppose invasion by intracellular microbes is the variety of mechanisms microbes use to escape, counteract or subvert the autophagy machinery [8, 9, 10]. Multiple factors are able to activate autophagy. Those include hypoxia, nutrient deprivation, oxidative stress, energy limitation, endoplasmic reticulum (ER)-associated stress, mitochondrial dysfunction or irradiation. Signaling pathways involved in resistance to infection can also initiate autophagy. This can be the case for NF-κB pathway regulators or DNA sensors [15, 16]. Other factors susceptible to activate autophagy include infection-induced GTPases, E3 ubiquitin ligases of the tripartite motif protein family (TRIMs) and the exocyst complex that regulates the interaction of exocytic vacuoles with the plasma membrane [17, 18, 19, 20]. Here, we review a number of recent studies that revealed an unexpected connection between components of the complement system and the functioning of the autophagy machinery. We shall see that some complement factors have the capacity to activate or modulate the autophagic response to infection by intracellular microbes.

## THE AUTOPHAGY PROCESS IN MAMMALS

Macro-autophagy, thereafter referred to as autophagy, is a multistep cellular process that relies on the engagement of dozens of factors encoded by highly conserved genes (Atg). At steady state, autophagy is maintained at a basal level by mechanistic target of rapamycin complex 1 (mTORC1) whose kinase activity controls the so called ULK1 kinase complex (Unc51-like kinase (ULK1)/ATG13/ATG101/FIP200). Perturbation of mTORC1 activates the ULK1 kinase complex causing the recruitment of the class III phosphatidylinositol 3-kinase (PI3K) vacuolar protein sorting (VPS)34 factor that promotes the formation of isolation membranes through the addition of phosphatidylinositol 3-phosphate (PI(3)P) onto membrane microdomains (initiation phase). Other factors contribute to (BECLIN1, VPS15 and ATG14L assembled in the Class III PI3K Complex I), or modulate (VMP1, AMBRA1, Bif1 and RUBICON), this step that is named the initiation phase [21, 22, 17]. Through engagement of the PI(3)P-binding factor WIPI2 and the recruitment of two ubiquitin-like conjugation systems, the isolation membrane extends to become the phagophore, a step named the elongation phase: on the one hand, ATG7 and ATG10 promote the formation of the ATG5-ATG12-ATG16L1 complex that is directed to the nascent phagophore via WIPI2 and on the other hand, ATG7 cooperates with ATG4B to convert the microtubule-associated protein, light chain 3 (MAP1LC3/LC3) factors into a form (LC3-I) prone to phosphatidylethanolamine (PE) addition in the presence of both the ATG3 factor and the ATG5-ATG12-ATG16L1 complex, leading to production of LC3-II. LC3-II indeed serves as a marker for the presence of autophagic membranes as it gets massively integrated into the elongating isolation membrane and stably persists until the phagophore evolves into a closed double-membrane vesicle that sequesters cytosolic cargoes and constitutes the autophagosome. LC3-I/II levels can be easily probed by Western blot-coupled SDS-PAGE and engineered versions of LC3 permit the quantitative analysis of autophagic activity through the numbering of fluorescent LC3 puncta by confocal microscopy. Along with GABARAP factors, factors of the LC3 type constitute the ATG8 family of core autophagy factors. Fully constituted autophagosomes become autolysosomes by fusing with lysosomes with, in some circumstances, an intermediate fusion step with endosomes, a step referred to as maturation. Autolysosomes represent the effective site of cargo degradation due to the catalytic microenvironment brought by the lysosomal vesicles [23, 24, 22, 17]. Autophagosome maturation involves multiples factors of different classes including Rab GTPases, cytosqueleton proteins, soluble N-acetylmaleimide-sensitive factor attachment protein receptors (SNARES), SYNTAXINs, membrane-tethering components of the homotypic fusion and vacuole protein sorting (HOPS) complex, endosomal sorting complexes required for transport (ESCRT) factors and the plekstrin homology domain containing adaptor PLEKHM1 [25]. The process is promoted by the BECLIN1-VPS34-VPS15-UVRAG complex (Class III PI3K complex II) and other factors including BIF-1. While autophagy is thought to be non-selective in the case of nutrient deprivation, the targeting of particular cargoes to autophagic degradation often involves the engagement of dedicated autophagy receptors that connect the cargoes to the growing phagophore by binding LC3-II via LC3-interacting regions (LIRs) domains and ubiquitin/lectin tags on cargoes via distinct specialized domains. This can be the case during the autophagic targeting of damaged organelles or of intracellular bacteria. Examples of well-studied autophagy receptors include optineurin, sequestosome 1 (SQSTM1/p62), nuclear dot 52 KDa protein (NDP52), NBR1, TRAF6-binding protein (T6BP/TAX1BP1) or NIX-BNIP3 [26, 27]. Interestingly, particular autophagy receptors (NDP52, Optineurin, TAX1BP1) are characterized by a duality of function as they are also involved in the occurrence of efficient autophagosome maturation [28, 29, 30].

## THE COMPLEMENT SYSTEM

The complement system represents an important arm of the innate immune system. It is made of dozens of soluble and membrane-associated proteins that can cooperate very rapidly to resist infection by various pathogens including bacteria, viruses, fungi and protozoa [31]. Soluble complement factors are massively produced in the liver and released in the blood. Proteins of the complement system are germline-encoded factors that react to microbial surface and facilitate microbe disposal by specialized phagocytes or directly trigger their lysis via lethal membrane damage. Concomitantly, complement factors facilitate the recruitment of other immune cell types by initiating inflammatory reactions. The complement system also modulates adaptive immunity through regulation of follicular dendritic cells and B cells for instance, contributes to the elimination/recycling of immune complexes and apoptotic cells and is instrumental for the homeostasis of multiple immune cell types [32, 33]. A central component of the complement system is the so-called C3 factor that is produced first as the pro-C3 form and is then subjected to processing while progressing through the secretory pathway that ultimately releases a complex made of a α and a β chain linked by a disulfide bond. Required for the activation of the complement system is the hydrolysis of circulating C3 by convertase enzymes that disrupt the thioester link leading to co-production of a short product called C3a and a larger one named C3b. C3b behaves as an opsonin that gets deposited onto target surfaces though formation of an amide or ester bond between its exposed active thioester and available amino-acid or carbohydrate moieties. Once opsonized, C3b and its potential byproducts can be recognized by adequate receptors present on the surface of various phagocytic cells [34]. Unlike C3b, C3a is an anaphylatoxin. It triggers local inflammation through activation of monocytes, endothelial cells, mast cells and neutrophils. Hence, the cleavage of C3 by C3 convertases translates into both pathogen targeting for physical elimination and localized inflammation. Once initiated, complement activation gets amplified by dedicated serine proteases while remaining under the control of membrane-associated and soluble regulatory factors [31, 35, 36, 37].

The so-called alternative, classical and lectin pathway are the three pathways capable of activating the complement system in an independent manner. The converging point of these pathways is the activation of C3. The alternative pathway is highly efficient in resisting microbial invasion. Under physiological conditions, minute quantities of C3 are naturally activated through spontaneous alteration of the intramolecular thioester bond leading to production of C3(H_2_O), a C3b equivalent that deposits on target surface [38]. If encountering healthy host cells, C3(H_2_O) is classically inactivated by regulatory factors such as Factors I and H. If it deposits onto abnormal cells or microbes instead, it recruits Factor B to assemble the pro-convertase C3(H_2_O)B that itself recruits Factor D and Factor P (properdin) to form the C3 convertase C3(H_2_O)Bb. As a consequence, regular C3b is produced that further deposits on the targeted surface and, via engagement of Factors B, D and P, produces the C3 convertase C3bBb [39]. This amplification effect promotes the generation of large amounts of C3b and therefore maximizes the rapid opsonization of the targets. The classical pathway involves the assembly of antibody-antigen complexes. IgM, IgG3 and IgG1 are the main antibodies interacting with the globular domain of the factor C1q via their Fc portion. Associated to C1q are the serine proteases C1r and C1s. Their activation causes the cleavage of the factor C4 into C4a and C4b with the latter binding covalently to targets in a C3b-like manner [40]. Subsequently, the factor C2 is recruited and cleaved by C1s to produce the protease C2a that along with C4b generates C4b2a, the main C3 convertase that reduces C3 into C3a + C3b within this pathway [41]. Within the lectin pathway, the C3 convertase production resembles that of the classical pathway with however an early step relying on the sensing of microbial oligosaccharides by lectins and the participation of lectin-associated proteases. Among host lectins involved are the collectin-LK and the mannan-binding lectin (MBL) that recognize carbohydrates and the ficolins 1-3 that can bind to acetylated groups on sugar moieties [42, 43]. Host lectins then associate with dimers of MBL-associated serine proteases (MASP-1 and -2). Upon engagement, MASP-1 gets auto-activated and cleaves MASP-2 that itself hydrolyses both C4 and C2 favoring the production of C4b2a, the C3 convertase already seen in the classical pathway [44, 45, 46]. Once C3b deposits on targets, recruitment of the Factor B promotes the formation of the pro-convertase C3bB that is converted into C3bBb by the Factor D. C3bBb, the C3 convertase of this pathway, is the functional equivalent of the C3 convertase C4b2a that operates in both the classic and lectin pathway. Again, the neo-production of C3b further amplifies the formation of C3bBb. Hence, both the classical and lectin pathway of complement activation involve the sensing of particular components on the target surfaces.

The final step of the response is the step of the target lysis. In contrast to the initiation phase, this terminal phase does not vary in its modality. It is dependent on the quantity of C3b that got associated with the target surface during the initiation phase. Subsequently to C3b recruitment, the C3 convertases C3bBb and C4b2a catalyze the formation of the C3bBb3b and C4b2a3b complexes endowed themselves with a convertase activity that targets the C3-related factor C5 [47, 48]. In this context, C5 gets degraded into C5a and C5b products allowing for the recruitment of the factors C6 to C9 by C5b to generate the C5b-9 complex, also named the membrane attack complex (MAC), on the targeted surface [49]. While C5a promotes localized inflammation, the MAC creates pores in biological membranes leading to the death of targeted microorganisms. Through binding to C3aR, C5aR1 and C5aR2 receptors on host cells, the anaphylatoxins C3a and C5a trigger various pro-inflammatory reactions such as oxidative burst, interleukin production, chemotaxis and production of histamine and leukotrienes [50, 51].

The corollary of the potent capacity of the complement system to cause microbe elimination through either internalization/degradation or MAC-mediated lysis is the requirement for a tight regulation to preamp damages to host cell/tissues. Multiple factors of the complement system are indeed directly involved in such a regulation. Collectively, they are named the regulators of complement activation (RCAs). RCAs can be either membrane-bound or soluble and not surprisingly, genetic alteration in RCA genes can be associated with marked pathologies [52, 53]. While the Factor H, the C1 inhibitor (C1INH) and the C4b-binding protein (C4BP) are the main soluble RCAs, the cell surface-associated RCAs include the membrane cofactor protein (MCP/CD46), the decay-accelerating factor (DAF/CD55) and the complement receptor-1 (CR1/CD35) and -2 (CR2/CD21). RCAs exert their regulatory function either through cofactor activity as illustrated by the cofactor effect CD46 exerts on the hydrolysis of the C3B/C4b opsonins by the serine protease Factor I or through decay-accelerating activity as exemplified by the dissociation of the catalytic region of the C3/C5 convertases by CD55. In some instances, RCAs can mediate both effects. Thus, Factor H concomitantly interferes with C3 convertases via a decay-accelerating effect and neutralizes C3b by serving as a cofactor for its reduction to the iC3b form by Factor I. It is remarkable to note that some microbes evolved the capacity to produce factors that promote the coating of their own surface with RCAs or to release factors that harbor biochemical properties resembling those of RCAs [54].

## NOVEL FUNCTIONS AND ACTION SITES FOR COMPLEMENT FACTORS

In the recent years, various observations brought support to the notion that the complement system not only contributes to immunity but is also involved in the regulation of multiple biological phenomena including mitosis, tissue/cell homeostasis, development, metabolism or tissue repair with some of such regulations being mediated by complement factors that are synthetized and secreted locally (reviewed in [33, 55]). In addition, recent findings also revealed that complement system factors can exert some functions within cells either directly in the cytosol or, within particular intracellular vesicles [56, 57, 33, 55, 58]. An interesting example is the intracellular effect that complement factors C3b and C4b can mediate after endocytosis of the microbe they were adsorbed to. In epithelial cells infected with the adenovirus, C3 factors attached to the virus get into contact with cytosolic components after exit from the endocytic vacuole, is capable of activating the mitochondrial antiviral signaling (MAVS)-related pathway and ultimately causes the production of pro-inflammatory cytokines via NF-kB engagement, protein-1 (AP-1) and interferon regulatory factor (IRF)-3-5-7 activation. At the same time, C3b can also direct the virus to the proteasome for degradation [59]. Activation of NF-kB via adsorbed C3b has also been observed for *S. Typhimurium* and the astrovirus 1 and coxsackievirus B3 non-enveloped viruses. In some instances, this activating effect on the NF-kB pathway generates only marginal effects because some microbes, such as enteroviruses, harbor enzymatic activity able to hydrolyze C3 present on their surface [59]. Besides reaching the cytosol after microbe internalization, functional complement factors can be found within fibroblasts, endothelial cells, epithelial cells or T cells due to endogenous synthesis [60, 61, 62, 63]. Unlike in other cells, the cleavage of intracellular C3 into C3a and C3b is mediated by the lysosomal hydrolase cathepsin-L within human resting CD4 T cells. In such cells, C3a has been found to favor cell survival via tonic modulation mTOR functions after binding to the C3a receptor (C3aR) on lysosomes [57]. Subsequently to T cell receptor (TCR)-mediated activation, the C3a-C3aR interaction gets relocated to the cell surface where it promotes a more stringent mTOR activation and initiates metabolic changes compatible with differentiation into effector lymphocytes [64]. Understanding the intracellular functions of complement factors now represents an expanding field of research. The notion of a “cell-intrinsic” complement system has been put forward to describe these functions and the question of whether the intracellular functions of complement may have evolved prior to extracellular functions has been raised [63, 58].

## THE HUMAN COMPLEMENT REGULATORY FACTOR CD46 RAPIDLY TRIGGERS AUTOPHAGY IN RESPONSE TO PATHOGEN SENSING

**Figure 1:**
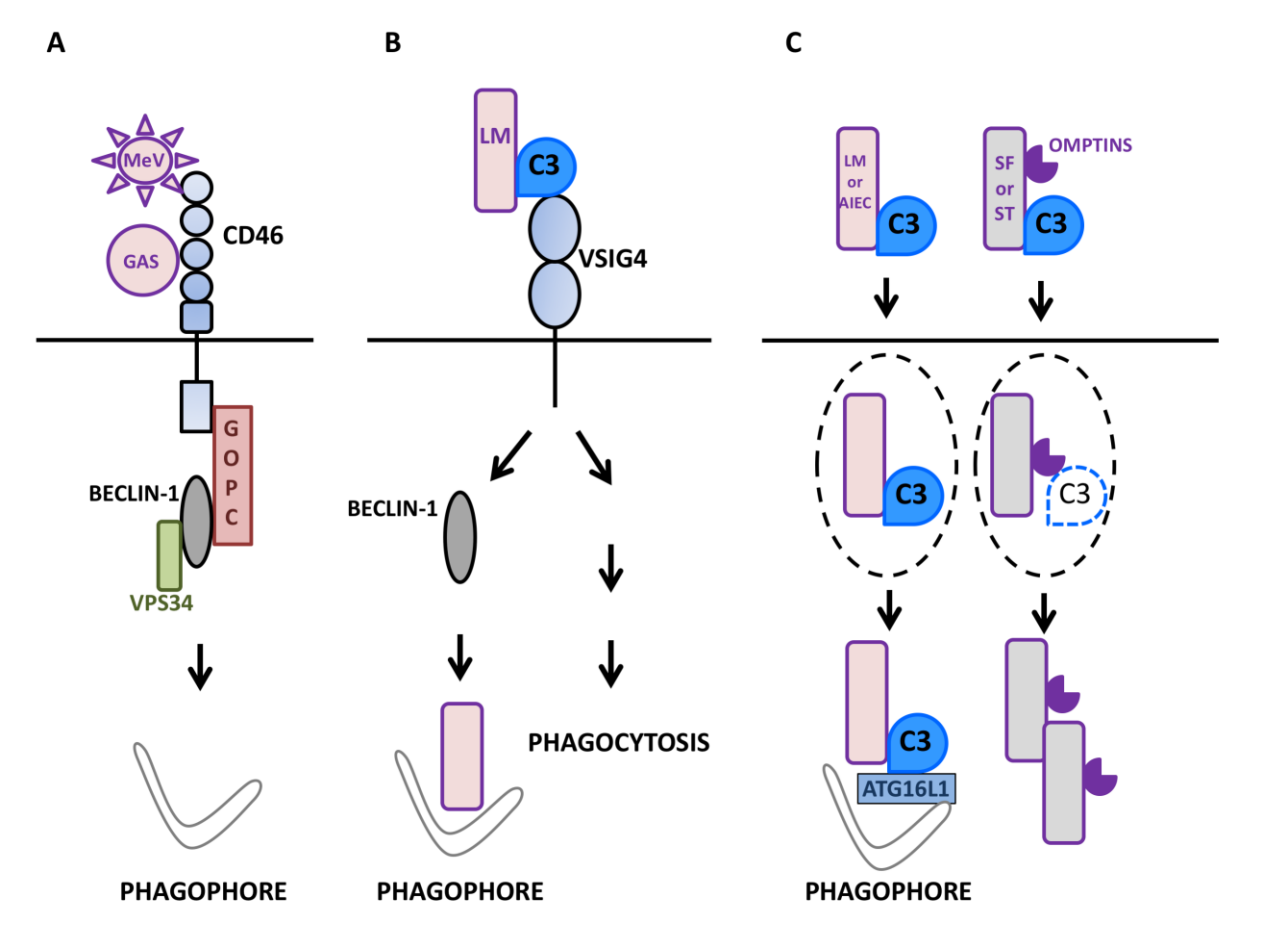
FIGURE 1: Autophagy induction by factors of the mammalian complement system in interaction with microbes. **(A)** Upon sensing of pathogens such as Measles virus (MeV) or group A *Streptococcus* (GAS), the regulator of complement activation CD46/MCP signals for autophagy induction in epithelial cells (depicted here by phagophore formation) by recruiting the BECLIN-1-VPS34 complex via the GOPC scaffold protein. **(B)** The macrophage surface receptor VSIG4/CRIg activates autophagy in a BECLIN-1-dependent manner in response to detection of C3 adsorbed onto *L. monocytogenes* (LM). Such an induction leads to the effective targeting of internalized bacteria to LC3-positive punctiform structures and then, lysosomal compartments. VSIG4/CRIg is also a positive regulator of phagocytosis in macrophages. **(C)** C3 deposited onto invading bacteria can induce autophagy by activating the autophagy machinery after reaching the cytosol of epithelial cells. Thus, after exiting their containing vacuole (dotted lines), C3-coated *L. monocytogenes* (LM) and *adherent-invasive Escherichia Coli* (AIEC) activate autophagy via the interaction of C3 with ATG16L1, one of the core autophagy factors. As a result, both bacteria are restricted by the induced autophagy. In contrast, other bacteria are able to escape such an antibacterial autophagy and multiply. For instance, C3-opsonized *S. flexneri* (SF) and *S. Typhimurium* (ST) are able to oppose autophagic restriction by lowering the level of the cytosolic C3-ATG16L1 interaction. This is due to their capacity to express proteolytic factors called Omptins that target C3 and reduce its amount deposited on the surface of bacterial cells.

The measles virus (MeV) is an enveloped RNA virus of the genus Morbillivirus of the Paramyxoviridae family. MeV entry into host cells requires interaction with a surface receptor. While clinical/virulent strains engage Nectin4 and CD150/SLAM to enter epithelial cells and immune cells respectively, attenuated/vaccinal strains use CD46/MCP to infect human cells [65]. CD46 is expressed on all nucleated cells and as indicated above, it acts as an important complement regulatory factor to avoid cell lysis by complement. By binding to C3b and C4b that deposit on cells, CD46 serves as a cofactor for the serine protease Factor I that hydrolyzes them leading to production of iC3b + soluble C3f and C4d + soluble C4c, respectively [66, 67]. Vaccinal/attenuated MeV strains rapidly activate autophagy during epithelial cell infection. This activation is a direct consequence of its interaction with CD46 and translates into both LC3-II formation and LC3-positive puncta formation with a maximum reached 1.5 h after infection [68]. MeV-induced autophagic activity then normalizes by 3h without the need for active inhibition. MeV infection associated autophagy is independent of viral protein synthesis as shown by its unperturbed induction in the presence of non-replicative MeV particles. The triggering of autophagy is indeed strictly related to the engagement of CD46 on the cell surface. For instance, the cross-linking of CD46 by the mean of monoclonal antibodies is sufficient to cause autophagy induction in human epithelial cells. With respect to the mechanism involved, CD46-induced autophagy relies on the engagement of the CD46-Cyt-1, but not -Cyt-2, intracytoplasmic splice variant [69] as demonstrated by the failure of vaccinal/attenuated MeV to activate autophagy in cells devoid of CD46-Cyt-1 [68]. The connection between CD46-Cyt-1 and autophagy triggering involves interaction with the class I PDZ portion of Golgi-associated PDZ and Coiled-Coil Motif containing (GOPC), a scaffold protein able to interact with the VPS34-BECLIN1 complex. Accordingly, silencing the expression of GOPC neutralizes the capacity of vaccinal/attenuated MeV to activate autophagy with no effect on the surface expression of CD46. It is worth emphasizing that the early burst of autophagy induced by CD46 engagement is strictly decoupled from the subsequent and sustained autophagic activity associated with MeV infection. In the latter case, autophagy is directly related to viral replication and in fact, is beneficial to the virus [70, 30]. Consistent with the key role for CD46 in early autophagy induction by vaccinal/attenuated MeV, virulent/clinical MeV strains that are independent of CD46 for infection do not trigger any signs of early autophagic activity, indicating that the CD46-Cyt-1-GOPC-VPS34-BECN1 complex axis constitutes the sole pathway for early autophagy induction by vaccinal/attenuated strains of MeV [71]. The CD46-Cyt-1-GOPC-VPS34-BECN1 complex pathway is also operating in autophagy induction during infection of human cells by GAS [68] and *Neisseria gonorrhoeae* [72] that both bind to CD46. As CD46 is also a receptor for various other pathogens such as the human herpes virus HHV-6, the BVDV pestivirus and adenoviruses B and D, the CD46-Cyt-1-GOPC-VPS34-BECN1 complex axis is likely to also trigger autophagy during infection by those microbes [73]. Thus, the observations made with the Morbillivirus MeV allowed for the identification of the complement regulatory factor CD46 as a pathogen sensor endowed with the capacity to activate autophagy in cells undergoing infection **(Fig.1A)**.

## THE ENGAGEMENT OF THE VSIG4 RECEPTOR BY C3b TRIGGERS AN EFFECTIVE ANTI-BACTERIAL AUTOPHAGY IN PHAGOYTIC ANTIGEN PRESENTING CELLS

V-set and immunoglobulin domain containing 4 (VSIG4/CRIg) is a surface receptor expressed on macrophages, monocytes, and dendritic cells [74, 75]. It is known to function as a C3b receptor involved in the efficient phagocytosis of bacteria, such as *Listeria monocytogenes,* coated with C3b. VSIG4 also participates in the efficient acidification of bacteria-containing vesicles through the modulation of the CLIC3 channel protein [76]. Recently, VSIG4 signaling was in fact found to also promote LC3 lipidation and structuration of LC3-positive puncta during infection of macrophage-like J774 cells by C3-coated *L. monocytogenes* [77]. Similar to CD46, the crosslinking of VSIG4 with specific monoclonal antibodies is sufficient to cause formation of LC3-II and puncta accumulation in THP1 or J774 macrophage-like cells. In contrast to CD46 engagement, however, the maximal level of LC3 lipidation was seen clearly latter: between 60 and 120 min after stimulation. Under such conditions, an increased phosphorylation of BECLIN1 was observed indicating that VSIG4 engagement alleviated BECLIN1 repression [76]. Such an autophagy induction was associated with a reduction in the level of the long-lived factor p62/SQSTM1 suggesting that the induced autophagy flux was complete. In macrophages infected with *L. monocytogenes*, internalized bacteria can escape phagosomes before they fuse with lysosomes by producing the phospholipase C and listeriolysin O virulence factors allowing them to access the cytosolic environment. Under conditions of VSIG4 cross-linking, *L. monocytogenes* could colocalize with LC3-positive punctiform structures suggesting that cytosolic bacteria were targeted by the autophagy machinery. Such a targeting was not seen when the autophagy machinery was mobilized through starvation as opposed to antibody-mediated VSIG4 engagement. Many of the bacteria that colocalized with LC3 puncta also colocalized with the lysosomal marker LAMP1, an effect that was sensitive to the autophagosome maturation blocker NH_4_Cl, suggesting that, in cells treated with anti-VSIG4 antibodies, *L. monocytogenes* was effectively directed to lysosomes via the autophagy machinery. When experiments were performed with THP1 macrophages devoid of the autophagy factor ATG5, *L. monocytogenes* was found capable of efficient proliferation with no detectable LC3 colocalization despite the antibody-mediated engagement of surface VSIG4 [77]. Of interest was the fact that VSIG4 signaling was associated with ubiquitination of cytosolic bacteria. Hence, the signaling event(s) associated with VSIG4 engagement induce an autophagic activity able to target *L. monocytogenes* to autophagic degradation. By using HeLa epithelial cells engineered to express VSIG4 it was observed that the lipidation of LC3 was markedly higher when cells were exposed to C3-coated *L. monocytogenes* as opposed to non-opsonized bacteria. In the latter situation, the level of LC3-II could be augmented by the co-presence of anti-VSIG4 antibodies. Such experiments conducted with modified epithelial cells demonstrated that no contribution specific to macrophages other than VSIG4 engagement was necessary to trigger autophagy. Consistent with data obtained with macrophage-like cells, extinction of the core autophagy factor ATG5 in VSIG4-HeLa cells resulted in the lack of LC3 lipidation and augmentation of *L. monocytogenes* multiplication, further indicating that VSIG4 engagement activated anti-bacterial autophagy. The capacity of VSIG4 signaling to oppose cytosolic bacteria growth by promoting autophagy was confirmed by taking advantage of Vsig4 deficient mice. In macrophages derived from Vsig4 deficient bone-marrow progenitors, the phagocytosis of C3-opsonized *L. monocytogenes* was lowered, LC3 lipidation and puncta formation were impaired, the polyubiquitination of cytosolic bacteria was limited and their proliferation was enhanced. Thus, the studies with primary macrophages nicely recapitulated the key observations made *in vitro* by using macrophage-like cell lines. Altogether, these observations demonstrated that through the engagement of the VSIG4 receptor, C3 adsorbed on *L. monocytogenes* can promote an autophagic response able to restrain the cytosolic growth of bacterial cells that escape phagosomes in professional phagocytic cells **(Fig.1B)**.

## THE C3-ATG16L1 INTERACTION ACTIVIATES ANTI-BACTERIAL AUTOPHAGY IN EPITHELIAL CELLS

As indicated above, once adsorbed on adenoviruses, C3 is able to activate the MAVS signaling pathways leading to secretion of pro-inflammatory factors [59]. It appears that C3 deposited on bacteria can also initiate anti-microbial defense in epithelial cells after reaching the cytosol. Searching for factors able to interact with human ATG16L1, Philpott and colleagues observed and validated that the C3 degradation fragment C3d was among its privileged partners [78]. These authors then examined the possibility that bacteria coated with C3 could impact the functioning of the autophagy machinery after accessing the cytosol of host cells via the C3-ATG16L1 interaction. As expected, C3-coated *L. monocytogenes* was more efficiently internalized by the phagocytic THP-1 cells relative to its non-manipulated counterpart. In addition, the presence of deposited C3 was associated with a lowered capacity of the bacteria to multiply in intestinal epithelial cells. In such cells, both ATG16L1 and LC3 displayed a stronger capacity to target cytosolic C3-coated *L. monocytogenes*. Under such conditions, C3-coated bacteria were found to efficiently co-localize with p62 and NDP52 autophagy receptors that engage interaction with ATG8 factors, with Galectin-8 that recognizes sugar moieties on damaged bacteria-containing vacuoles and with ubiquitin that binds to cytosolic bacterial cells, strongly suggesting that C3 opsonization of *L. monocytogenes* translated into an enhanced targeting by the autophagy machinery of epithelial cells. As a result, the resistance to the bacteria was augmented as illustrated by the C3-dependent restriction of cytosolic *L. monocytogenes* in ordinary epithelial cells but not in their counterpart that lacked ATG16L1. It was remarkable to observe that in complementation experiments, the autophagic restriction of C3-opsonized *L. monocytogenes* could not be restored by re-expression of the 300A variant of ATG16L1 that is associated with Crohn's disease (CD) [79]. The enhancing effect of C3 opsonization on anti-bacterial autophagy was indeed not restricted to *L. monocytogenes* as adherent-invasive *Escherichia coli* (AIEC), bacteria often found in expansion in patients with ileal forms of CD [80] and sensitive to autophagic degradation [81], could also be restricted by autophagy in an C3- and ATG16L1-dependent manner. Hence, by engaging ATG16L1 in the cytosol of host epithelial cells, C3 adsorbed on invasive bacteria efficiently promotes their restriction by autophagy. The anti-bacterial potential of this phenomenon is best emphasized by the observation that some bacteria evolved means to oppose the C3-ATG16L1 interaction. Thus, although C3-opsonized *Shigella flexneri* appears well targeted by LC3 and ATG16L1 when it accesses the cytosol of epithelial cells, its expansion is indeed poorly restricted. Such an escape relies on the ability of *S. flexneri* to shed the C3 factor with a majority of bacterial cells being C3-negative four hours after infection. The C3 shedding requires no contribution from host cells as it can be observed, for *S. flexneri* but not *L. monocytogenes,* during expansion in regular media *in vitro*. Omptins are proteolytic factors found at the level of the outer membrane in gram-negative *Enterobacteriaceae* [82]. LcsP is one of such enzymes that is found in *S. flexneri* [83] and was therefore examined for its capacity to cause C3 shedding. *S. flexneri* lacking lcsP had a clearly reduced capacity to lose C3 *in vitro* and to replicate in the cytosol of host cells. This cellular restriction was dependent on C3 in ordinary, but not ATG16L1 deficient cells in agreement with the notion that lcsP does promote the elimination of C3 from *S. flexneri*. In the same line, the omptin PgtE expressed by *S. Typhimurium* and capable of cleaving C3 [84, 85] could promote C3 shedding by Salmonella cells: *S. Typhimurium* lacking PgtE inefficiently shed C3 and once C3-opsonized, became sensitive to C3-dependent restriction in ordinary, but not ATG16L1 deficient cells. Hence, by catalyzing the shedding of C3, omptin enzymes endowed both *S. Typhimurium* and *S. flexneri* with the capacity to escape the pro-xenophagic role of deposited C3 in host epithelial cells. Interestingly, both lcsP and PtgE partially controlled the shedding of C3 suggesting that additional enzymes are likely to be involved in this process **(Fig. 1C)**. In a mouse intra-gastric model of infection with *L. monocytogenes* [86], it was observed that *L. monocytogenes* caused the production of C3 in intestinal mucosa, showing that C3 can be in close proximity to intestinal cells in the course of infection. In contrast with lymphoid organs where it was stable, the load of bacteria was found clearly augmented in the colon and caecum from C3 deficient mice. The extent of Listeria accumulation was indeed comparable to that observed in mice with ATG7 deficiency restricted to intestinal epithelial cells. Also of interest was the observation that despite an unperturbed level of ATG16L1, the expression level of both LC3 and ATG7 core autophagy factors was lowered after Listeria infection, indicating that the autophagy machinery was perturbed in C3-deficient mice. The bacterial burden seen in C3 deficient mice could be brought back to the level of that seen in normal mice by simply administrating the autophagy activator rapamycin to animals confirming that the anti-Listeria autophagic response of C3 deficient intestinal cells was readily altered. Hence, C3 is efficient at enhancing the anti-Listeria autophagic response in the intestine of infected mice. Besides the C3-ATG16L1 interaction, this contribution might involve a transcriptional modulation of the LC3 and Atg7 genes and perhaps, other factors that remain to be examined.

Interestingly, endogenously produced cytosolic C3 is also capable of influencing autophagy through interaction with ATG16L1. For instance, human pancreatic beta cells were shown to produce C3 that positively regulates autophagy via its interaction with ATG16L1 thereby contributing to cell homeostasis and stress resistance [87]. The endogenously produced intracellular C3 corresponds to its precursor form as it does not access the classical secretory pathway. As a consequence, the intracellular C3-ATG16L1 interaction appears to require neither C3 glycosylation nor the production of the processed form of C3 (the α/β two chains form of C3). Whether the cytosolic environment of mammalian cells contains C3-convertase-like activity able to generate C3b that could deposit onto invading bacteria and enhance anti-bacterial autophagy as in the case of C3-coated Listeria is unknown. Collectively, the available results suggest that the C3-ATG16L1 interaction is capable of modulating autophagy not only at the initiation step but also at the level of autophagosome maturation. How these modulations take place and the detail of their regulation in various cell types remain to be investigated. Of interest is the fact that unlike the ATG16L1-native C3 interaction, the interaction of ATG16L1 with cleavage-products of C3 (C3b, iC3b, C3d, C3c) is very poor *in vitro* [87]. Hence, the cleavage of intracellular C3 by endogenous enzymes could possibly represent a mean to adjust the effects of the C3-ATG16L1 interaction. Pools of cytosolic C3 can be observed within several cell types such as cells of the immune system, fibroblasts epithelial cells and endothelial cells. The production of cytosolic C3, the conditions involved in the formation of C3 stores and the requirement for their persistence in distinct cell types remain to be fully characterized. Within CD4 T lymphocytes, it was noticed that the hydrolase cathepsin L is able to cleave C3 in order to generate C3a that positively regulate cell persistence through tonic activation of mTOR. Whether regulation of the autophagy pathway contributes to this pro-survival outcome deserves further investigation.

## THE ANCIENT IMMUNE SIGNALING PATHWAY Mcr/Draper MODULATES AUTOPHAGY IN DROSOPHILA

**Figure 2:**
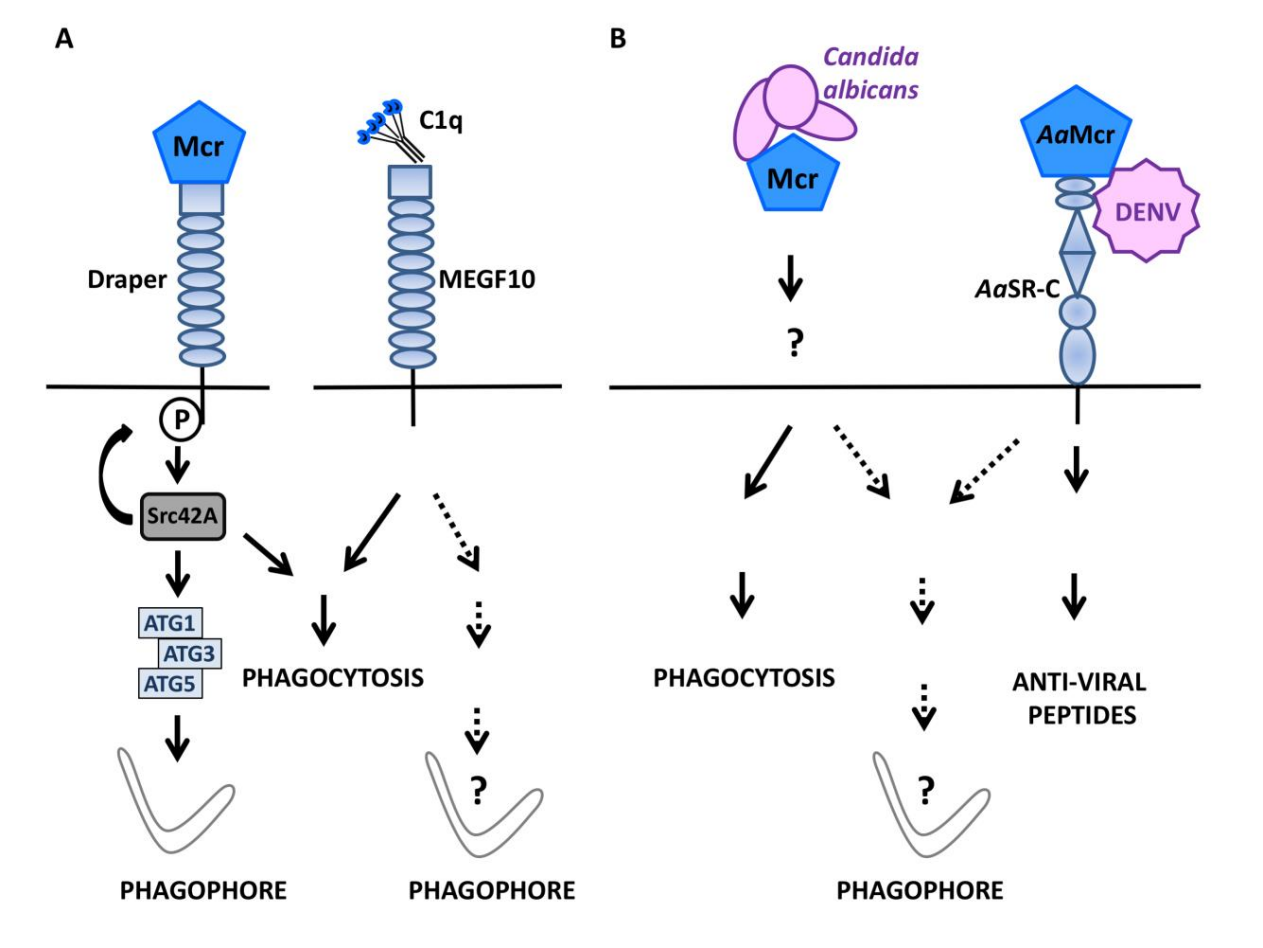
FIGURE 2: Macroglobulin complement related factor (Mcr) in antimicrobial defense and autophagy. **(A)** During *D. melanogaster* development, the complement-like opsonin Macroglobulin complement related/TEPVI (Mcr), that is highly similar to mammalian complement factor C3, can initiate autophagy in both epithelial cells and macrophages by engaging Draper, a Src42A protein kinase-coupled, EGF-like-repeat-containing receptor known to signal for phagocytosis (left). Whether Multiple EGF-like-domain Factor 10 (MEGF10), a mammalian ortholog of Draper that mediates phagocytosis in response to C1q binding, can also signal for autophagy induction is unknown (right). **(B)** Mcr mediates the selective phagocytosis of *Candida albicans* in *D. melanogaster* (left) and, along with the scavenger receptor-C (*Aa*SR-C) is involved in initiating the production of anti-viral peptides in response to the Dengue virus (DENV) in *Aedes aegypti* (*Aa*Mcr)(right). Whether pathogen sensing by Mcr (with a role for Draper in the case of *D. melanogaster*?) can activate anti-microbial autophagy in insects remains to be studied.

During *Drosophila melanogaster* development, hormones promote cell death in certain organs such as larval salivary glands. During this phase, active caspases and autophagic activity are both involved in the disposal of cell debris [88]. Studying the regulation of autophagy during this process, Lin *et al.,* [89] examined a possible role for Draper, an ancient immune receptor that is a member of the epidermal growth factor (EGF)-like-repeat-containing receptor family and a *D. melanogaster* orthologue of cell death abnormality protein-1 (CED-1) in *Caenorhabditis elegans*. Draper contains an intracellular portion that signals for phagocytosis in response to altered self [90, 91], is known to act upstream of genes of the autophagy machinery and is required for autophagy associated with the developmental remodeling of salivary glands [92]. Indeed, the external portion of Draper turned out to be mandatory for salivary gland remodeling suggesting that an extracellular cue is able to activate Draper-activated autophagy. With this respect, a role for macroglobulin complement-related (Mcr/TEPVI), a factor related to the thioester-containing protein family, was investigated because its expression is detected in salivary glands and its level increases in the presence of ecdysone. Mcr is among the best-described complement-like opsonins in *D. melanogaster* where it represents an important defense factor against *Candida albicans* infection [93]. Lin *et al.* found that *mcr* invalidation in salivary glands caused the aggregation of vacuolated cellular fragments that closely resembled those observed upon autophagy blockade via Atg13 knockdown. Enforcing autophagy via Atg1 overexpression could compensate for such a degradation defect, pointing to an important role for Mcr upstream of Atg1 in the autophagy process involved in salivary gland cell disposal. Mcr is in fact mandatory for salivary gland-associated autophagy as shown by the scarcity of Atg8a punctiform structures and an increased level of Ref(2)P, the *D. melanogaster* ortholog of p62/SQSTM1, in salivary glands lacking Mcr. Remarkably, exogenous Mcr is capable of influencing the formation of Atg8a-positive puncta in wild type and Mcr deficient cells to a similar extend, indicating that Mcr can regulate autophagy in a non-cell autonomous manner. *In vivo*, the cumulative mutations of *mcr* and *draper* caused a more marked degradation defect than either mutation considered individually. This phenotype was associated with a rarefaction of Atg8 puncta and reverted upon Atg1 ectopic expression. Of interest was the fact that a constitutively active version of Src42A, the enzyme responsible for Draper phosphorylation and that is required for degradative activity during salivary gland development, could compensate for the defect imposed by Mcr deficiency. Hence, Mcr functions upstream of draper to signal for autophagy induction. In *D. melanogaster* embryo, the Draper-Src42A module also participates in macrophage migration to damaged epithelium **(Fig. 2A)**. In the absence of Mcr, the migration of macrophages is reduced and most of them display a low level of Atg8a puncta formation because epithelial cell production of Mcr along with Draper expression by macrophages, are necessary for autophagy induction. The exogenous provision of Mcr is sufficient to activate autophagy in embryonic macrophages *in vitro* in a Draper and Atg1/3/5 dependent manner. Thus, in *D. melanogaster*, interaction of the complement-like opsonin Mcr with the EGF-like-repeat-containing receptor Draper can mobilize the autophagy machinery in a non-cell autonomous fashion. This pathway is instrumental for the regulatory role of autophagy both in the remodeling of salivary gland during development and in macrophages involved in the resolution of epithelial cell death in the embryo. The productive engagement of Draper by Mcr for phagocytosis induction and the known role of Mcr in immune response to fungi [93] and flaviviruses [94] in insects suggest an ancient origin for the Mcr-Draper axis. The observations by Lin *et al.* [89] now reveal that this ancient immune signaling axis can function in the regulation of autophagy in the remodeling of tissues during development **(Fig. 2B)**. Phylogenetically, it would be interesting to determine whether Multiple EGF-like domain Factor 10 (MEGF10), a mammalian factor related to Draper and serves as a receptor for the C1q complement factor [95] can be connected to autophagy induction pathways.

## CONCLUDING REMARKS

A substantial corpus of recent observations indicates that besides their immune functions, complement system factors can regulate various cellular functions in both physiological and pathological settings. Factors of the complement system are indeed capable of directly promoting autophagic activity. For instance, cell surface receptors belonging to the complement system have the capacity to trigger anti-bacterial autophagy response as illustrated by the human complement regulatory factor CD46 that induces a marked burst of autophagy in epithelial cells in response to virus/bacteria sensing. In that case, autophagy induction relies on the CD46-Cyt1 isoform that recruits the GOPC factor leading to engagement of the VPS34-BECLIN1 complex. While CD46 interacts on an *in cis* mode when engaged in the neutralization of C3b, the VSIG4 receptor binds to C3b *in trans* to facilitate the endocytosis of C3b-coated Listeria by phagocytes. Although the detailed intermediate steps and factors involved remain to be identified, it is established that VSIG4-mediated activation of autophagy also involves the recruitment of BECLIN1.

A link between activation of the autophagy machinery and engagement of complement-related factors can also be observed in insects. Mcr, a drosophila factor related to C3, can initiate autophagy in epithelial cells of the developing salivary gland in both an autocrine and paracrine manner to regulate the homeostasis of the tissue. Via modulation of autophagy, Mcr can also regulate the migration of phagocytic cells to inflammatory sites during embryogenesis. Autophagy activation by Mcr involves the ancient microbe-sensing axis called the Draper-Src42A pathway. The mammalian ortholog of Draper is MEGF10 that is capable of interacting with C1q. Hence it would be interesting to examine whether the C1q-MEGF10 interaction can influence developmental processes and/or immune responses to microbial infection in mammalian cells via autophagy induction/modulation. Indeed, a modulatory effect of C1q on epithelial cell autophagy has been reported. It involves the interaction of C1q with the metalloproteinase ADAM 28 and disintegrin and appears to influence susceptibility to cell death [96]. It remains to be investigated whether other membrane-associated complement factors or complement factors interacting with surface receptors are able to regulate autophagy. In addition, it will be interesting to examine to which extend pathogens may have evolved means to oppose/escape, autophagy induction by components of the complement system.

A truly unanticipated finding was the capacity of C3 deposited onto bacteria to promote anti-bacterial autophagy via direct interaction with the core autophagy factor ATG16L1 after internalization. This enhancing effect on autophagy translated into an ameliorated restriction of bacteria as demonstrated by the altered response to Listeria infection in mice lacking C3. Of note, hydrolytic reactions that degrade C3 deposition on some bacteria allow them to escape the enhancing effect of the C3-ATG16L1 interaction on anti-bacterial autophagy. With respect to the cytosolic C3-ATG16L1 interaction and its role in anti-microbial autophagy, it remains to be determined whether endogenously produced C3 than can operate in maintaining homeostasis of cells such as pancreatic beta cells, can get adsorbed onto invading cytosolic bacteria and promote their restriction by the autophagy machinery. Retrospectively, it would be also interesting to determine whether C3 deposited on viruses such as adenovirus [59] can activate autophagy via ATG16L1 engagement once it exited the endocytic vacuole.

Among questions raised by the observation that factors of the complement system can regulate the autophagic activity is the issue of whether autophagy itself can in turn regulate the intracellular functions of complement factors. Possibly, the autophagy machinery could regulate the intracellular function of complement factors at large via their specific degradation or via degradation of factors necessary for their production in the case of complement factors produced intracellularly. Such regulatory effect would be somehow reminiscent of that involved in the regulation of the inflammasome by the autophagy machinery [97]. Finally, it remains also to be studied whether complement factors present intracellularly can interact with either a restricted or a larger set of the core factors involved in the autophagy flux and what are the functional consequences both at steady state and under conditions of infection.

Multiple observations now indicate that factors of the complement system mediate intracellular functions. It has been suggested that such phenomena could represent the manifestation of a cell-intrinsic complement system for which the term of “complosome” has been coined. As evoked above, one may ask whether such intracellular functions indeed co-evolved with the extracellular functions of the complement system or whether they could have even preceded it [98, 63, 58]. Regardless of the right answer, it seems fair to state that the relationship between autophagy and factors of the complement system, as well as the impact of this relationship on the defense against microbes, are very likely to have emerged early during evolution.

## Abbreviations:

ATG: – autophagy-related factor;
CD4 T: – mature T lymphocytes of the CD4 lineage;
C3aR: – C3a receptor;
C5aR: – C5a receptor;
EGF: – epidermal growth factor;
GAS: – group A Streptococcus;
GOPC: – Golgi-associated PDZ and Coiled-Coil Motif containing;
GTPases: – guanosine triphosphate hydrolase;
LC3 (MAP1LC3): – microtubule-associated protein 1-light chain 3;
Mcr: – macroglobulin complement-related;
mTORC1: – mammalian target of rapamycin complex 1;
MAC: – membrane attack complex (C5b-9);
MASP: – MBL-associated serine proteases;
MAVS: – mitochondrial antiviral signaling;
MBL: – mannan-binding lectin;
MCP: – membrane cofactor protein (CD46);
MEGF10: – multiple epidermal growth factor-like domain factor 10;
MeV: – Measles virus;
NF-κB: – nuclear factor-kappa B;
PI(3)P: – phosphatidylinositol 3-phosphate;
RCA: – regulators of complement activation;
ULK1: – Unc51-like kinase;
VPS: – vacuolar protein sorting.
